# Evaluation on the Efficacy of Farrerol in Inhibiting Shoot Blight of Larch (*Neofusicoccum laricinum*)

**DOI:** 10.3390/plants13213004

**Published:** 2024-10-28

**Authors:** Evaristo A. Bruda, Rui Xia, Ruizhi Zhang, Haoru Wang, Qi Yu, Mengyao Hu, Feng Wang

**Affiliations:** 1 Key Laboratory of Alien Forest Pest Detection and Control-Heilongjiang Province, College of Forestry, Northeast Forestry University, Harbin 150040, China; bruda.risto@gmail.com (E.A.B.); xiarui@nefu.edu.cn (R.X.); zhangruizhi@nefu.edu.cn (R.Z.); wanghaoru506@163.com (H.W.); yinr@nefu.edu.cn (Q.Y.); mengyaohu@nefu.edu.cn (M.H.); 2 Key Laboratory of Sustainable Forest Ecosystem Management-Ministry of Education, College of Forestry, Northeast Forestry University, Harbin 150040, China; 3 State Key Laboratory of Tree Genetics and Breeding, College of Forestry, Northeast Forestry University, Harbin 150040, China

**Keywords:** antifungal activity, disease resistance, gene expression, metabolite profiles, secondary metabolites

## Abstract

*Neofusicoccum laricinum* is the causal agent of larch shoot blight, a fungal disease affecting several species of larch. It causes severe damage, including stunting and mortality. This study aims to address the severe impact of larch shoot blight by evaluating the effect of farrerol on the inhibition of *Neofusicoccum laricinum* in *Larix olgensis*. We used LC-MS/MS and weighted gene co-expression network analysis to investigate farrerol’s effects on *Neofusicoccum laricinum* and identify associated genes in resistant and susceptible larch. Our study identified significant differences in metabolite profiles between resistant and susceptible cultivars, with higher concentrations of farrerol showing complete inhibition of *N. laricinum.* Additionally, specific genes associated with farrerol content were up-regulated in resistant larch. Farrerol at higher concentrations completely inhibited *N. laricinum*, showing a strong correlation with increased disease resistance. This research suggests that farrerol enhances disease resistance in larch and provides a foundation for developing disease-resistant larch varieties based on antifungal metabolite traits.

## 1. Introduction

Larch (*Larix*) species are deciduous conifers of the Pinaceae family, widely distributed in the northern hemisphere where permafrost and seasonally frozen soils occur [1,2,3,4]. Since 1999, the Chinese government has decided to reforest farmland (<25° slope) and degraded areas (Natural Forest Conservation Program [NFCP]) and has used larch in Northeast China to increase forest cover [5]. This species with heterophyllous shoots has been intensively planted [2]. As a native species, *Larix* is primarily distributed in northern Hebei, Shanxi, Qinling, Shaanxi, north-eastern Inner Mongolia, southern Gansu, western and south-western Sichuan, and north-western Yunnan. The growing stock of *larix* species in China reached about 1200 million m^3^, according to the Global Forest Resources Assessment (FRA) report [6]. Despite its high environmental adaptability, cold tolerance, rapid growth rate, and robust wood properties, *Larix* is susceptible to various biological and physical stressors. Along with exposure to low temperatures [7], larch shoot blight is another significant stressor affecting these species [8].

Larch shoot blight represents a significant threat to forest ecosystems, with a considerable impact on larch plantations [9]. The disease was first identified in 1938 in Hokkaido, Japan [10]. The pathogen was identified as *Neofusicoccum laricinum* (*Physalospora laricina* Sawada, 1950; *Guignardia laricina* Yamamoto and Kazuo, 1961; *Botryosphaeria laricina* Shang Yanzhong, 1987) [9,11]. The areas surrounding south-eastern Liaoning, eastern Jilin, most of Heilongjiang, and north-eastern Inner Mongolia were found to be moderately and highly affected by larch shoot blight [12]. Based on the fact that larch shoot blight is a host-dominant disease, the only viable solution is to breed resistant varieties. Nevertheless, the current lack of effective technology and standards for the breeding of resistant larch varieties represents a significant challenge. In our long-term control practice, we found that certain larch monocultures exhibit resistance to *N. laricinum*. Therefore, large-scale cultivation of resistant larch could significantly improve the prevention and management of *N. laricinum* through widespread cultivation of resistant larch. Consequently, there is an urgent need to elucidate the molecular mechanisms underlying disease resistance in different resistant monocultures. Further research on secondary metabolites against disease in larch will assist in the breeding of disease-resistant varieties. Many secondary metabolites are not only direct fungistatic compounds but also serve as indicator compounds for screening disease-resistant larch.

Plants have evolved a remarkable array of structural, chemical, and protein-based defenses to detect and prevent the establishment of invasive organisms before they can cause extensive damage [13,14]. Many plant species produce a wide variety of secondary metabolites, including alkaloids, terpenoids, flavonoids, phenolics, sulfur compounds, and quinones, in response to pathogens [15,16]. The functions of secondary metabolites in plant defense include deterrence, UV resistance, pigmentation, disease resistance, stimulation of nitrogen-fixing nodules, toxicity, and serving as a precursor to the physical defense system [13,14]. As a naturally occurring flavanone, farrerol has the same core structure as naringenin but contains two additional methyl groups on ring A [17]. It has been documented that farrerol possesses several biological activities such as antioxidant, anti-inflammatory, and antibacterial effects [17,18]. Farrerol possessed potent inhibitory activities against hyphal growth of *F. oxysporum* f. sp. *niveum*, *C. gloeosporioides*, *P. italicum*, *R. solani*, *F. oxysporum* f. sp. *Cubenserace*, and *P. melonis* [17,18].

The antifungal mechanism of farrerol involves the deformation of fungal hyphae, including increased branching, disruption of fungal cell membranes, and thinning and hollowing or splitting of the mycelia [18]. Despite the importance of farrerol in inhibiting various pathogenic fungi, there are still gaps in understanding its mechanism and efficacy in *Larix*, especially in China, where these species are vital in forest ecosystems. Consequently, this study aims to fill these gaps by analyzing the efficacy and mechanism of farrerol in combating larch shoot blight. This study contributes to the identification of disease-resistant cultivars that are capable of withstanding larch shoot blight following inoculation. Furthermore, it presents a promising avenue for enhancing disease resistance that warrants comprehensive investigation through the use of natural fungicides as well as the genetic modules that underlie this mechanism.

## 2. Results

### 2.1. Identification of Resistant and Susceptible Larch to N. laricinum 

**Figure 1:**
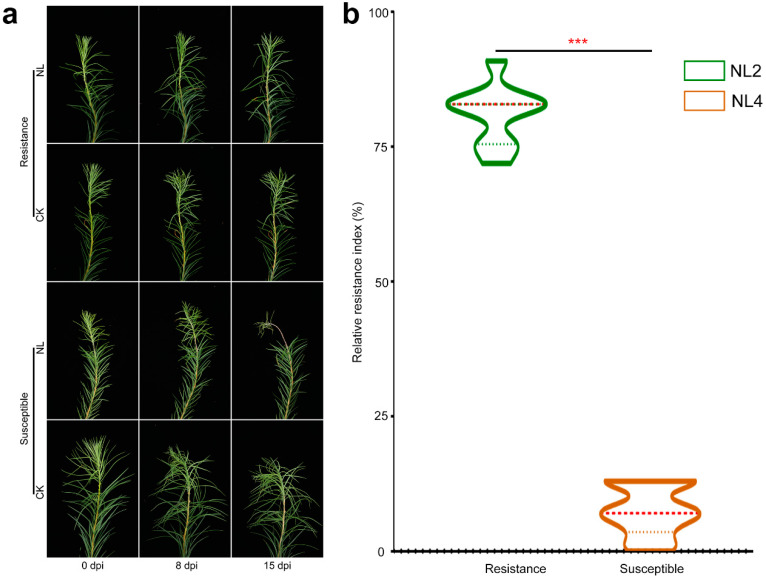
Assessment of resistance in larch to larch shoot blight. (**a**) Symptoms of NL2 and NL4 annual shoots inoculated with *N. laricinum*. (**b**) Analysis of differences in relative disease resistance indices of NL2 and NL4 (*** *p* < 0.001), green color represent resistant NL2 and Orange color represent sensitive NL4.

Artificial inoculation tests revealed significant differences in disease resistance among various larch monocultures. The response of the larch trees following inoculation was evaluated based on the observation of several symptoms, including dehydration, wilting, needle shedding, color changes in the branches and stems, and the size of the lesions at the inoculation sites. Larch NL2 and larch NL4 were screened for resistance and susceptibility to larch shoot blight disease. After 8 days of indoor artificial inoculation with *N. laricinum*, resistant and sensitive larch showed distinctly different symptoms. In the resistant larch exclusive few needles at the top of the branches changed color from green to yellow-brown, without apparent needle shedding in the upper part of the branch. The control branches of resistant NL2 inoculated with ddH_2_O remained green without shedding needles (Figure 1a). In contrast, the branch of sensitive NL4 showed severe symptoms in inoculated branches. The control sensitivity branches remained green without shedding needles. The inoculated sensitive NL4 branches also displayed dehydration, wilting, and shedding of needles and leaves, and the inoculated areas changed from yellow-green to black-brown. The average relative disease resistance index for the resistant was 81% compared to 7% for the sensitive monocultures, indicating a statistically significant difference in resistance (*p* < 0.001) (Figure 1b).

### 2.2. Identification of 16 Metabolites in Resistant Larch

**Figure 2:**
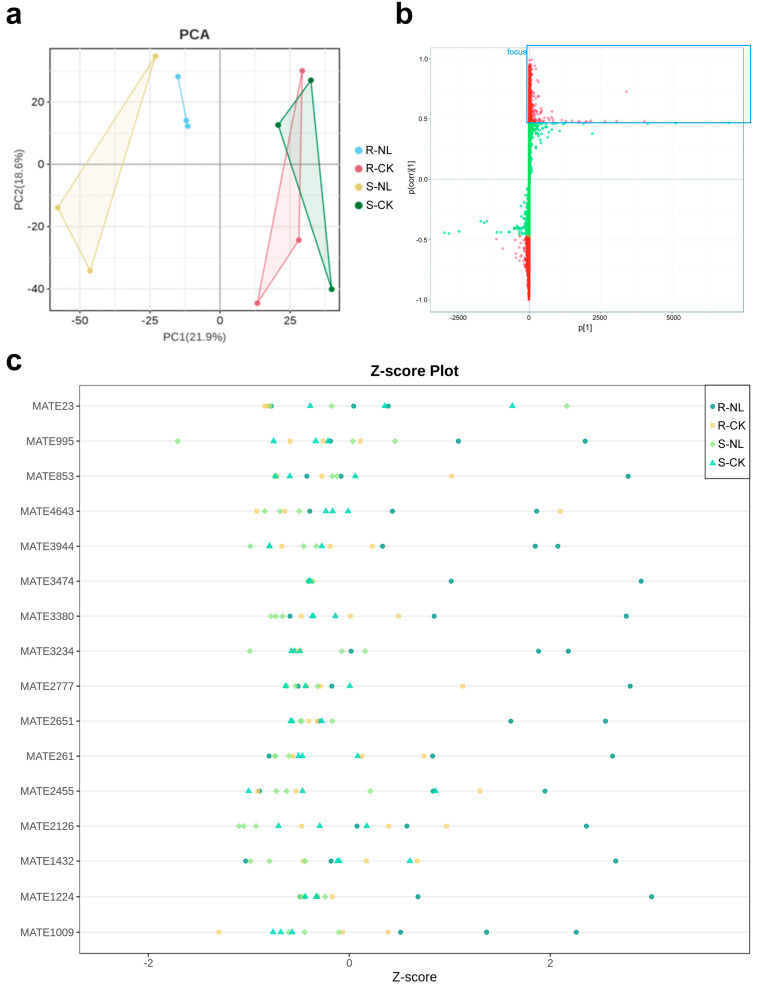
Metabolome analysis of resistant larch and susceptible larch. (**a**) Principal component analysis of metabolome data for NL2 and NL4 inoculated with *Neofusicoccum laricinum* and control groups (CK, mock inoculation with ddH_2_O). (**b**) S-plot of VIP values of metabolites for NL2 and NL4 inoculated with *Neofusicoccum laricinum* and CK groups. (**c**) Z-score of differential metabolites for NL2 and NL4 inoculated with *Neofusicoccum laricinum* and CK groups.

Non-targeted metabolic assay testing was conducted on four experimental groups for disease resistance and disease sensitivity. The treatment group was inoculated with *N. laricinum* for 8 days, while the control group was inoculated with ddH_2_O to simulate disease resistance and sensitivity. A total of 4746 metabolites were identified. To test for overall and within-group differences in the samples, the Orthogonal Partial Least-Squares Discriminant Analysis (OPLS-DA) model was employed to calculate OPLS-DA scores for disease resistance versus sensitivity (Figure 2a). The horizontal axis represents overall differences, while the vertical axis illustrates within-group differences. As illustrated in Figure 2a, the disease-resistant and disease-sensitive metabolites exhibited a distinct separation trend. The replacement test for the model yielded a parameter R^2^Y of 0.999, with an R^2^Y value close to 1, indicating that the model explains more data for the two classifications and more differences between groups. Additionally, Q^2^ was 0.455. The Q^2^ value indicates the predictability of the model, and a value greater than 0.4 indicates that the model is valid. In summary, the model was not overfitted; the VIP values calculated by the model could be used to screen for differential metabolites; there was little difference between good clustering within the disease-resistant and disease-sensitive groups; there were clear differences in metabolite composition between groups; and the metabolite compositions between the groups were significantly different.

To further screen for specific differential metabolites and measure the changes in the relative content of metabolites before and after inoculation with *N. laricinum* for disease resistance and disease sensitivity, respectively, at the same level, comparative analyses were carried out using *t*-tests for *p*-values, VIP values, and FC values to assess the relative content of metabolites. The S-plot derived from the OPLS-DA model was also utilized. The model demonstrated that there were notable differences in metabolites between disease resistance and disease sensitivity. The closer the metabolites are to the upper right and lower left corners, the more pronounced the difference is, and the red dot in the upper right corner of the figure indicates a VIP value of ≥1 (Figure 2b). A total of 1089 metabolites with significantly increased relative content were identified according to the established criteria. Of the 1089 metabolites, 743 exhibited a significantly increased relative content before and after inoculation with the disease-resistant strain, while 548 demonstrated a significantly increased relative content before and after inoculation with the disease-sensitive strain. Additionally, 541 differential metabolites exhibited significantly increased content observed exclusively in the disease-resistant samples, while the content in the disease-sensitive samples was either decreased or unchanged. Only 16 compounds were highly concentrated in disease resistance compared to susceptibility, and the levels increased significantly after inoculation with *Neofusicoccum laricinum* (Table 1). Based on the relative contents of the 16 differential metabolites, they were converted into standard scores (Z-scores). The graphical results were consistent with the above analyses (Figure 2c), thus confirming the selection of 16 differential metabolites, including farrerol, for subsequent correlation analyses.

**Figure 3:**
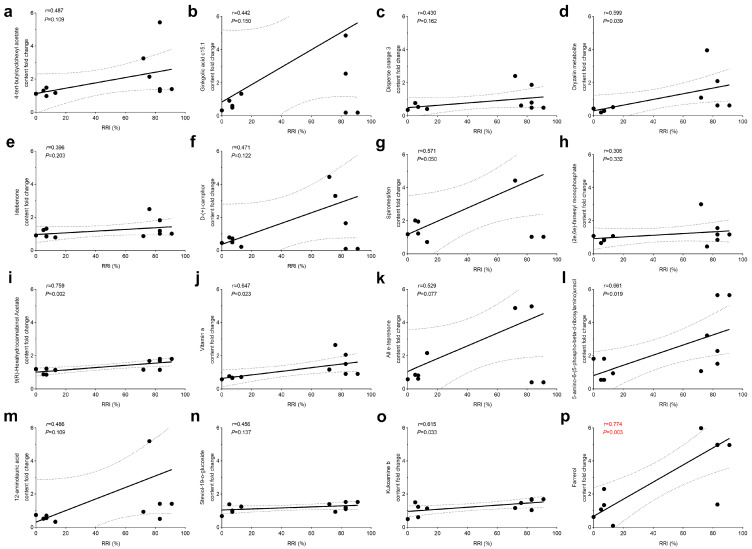
Correlation analysis between the relative content of selected differential metabolites and the relative resistance index of larch. (**a**) Correlation analysis between content fold chang of 4-tert-butylcyclohexyl acetate and RRI. (**b**) Correlation analysis between content fold chang of Ginkgolic acid c15:1 and RRI. (**c**) Correlation analysis between content fold chang of Disperse orange 3 and RRI. (**d**) Correlation analysis between content fold chang of Oryzalin metabolite and RRI. (**e**) Correlation analysis between content fold chang of Idebenone and RRI. (**f**) Correlation analysis between content fold chang of D-(+)-camphor and RRI. (**g**) Correlation analysis between content fold chang of Spiromesifen and RRI. (**h**) Correlation analysis between content fold chang of (2e,6e)-farnesyl monophosphate and RRI. (**i**) Correlation analysis between content fold chang of 9(R)-Hexahydrocannabinol Acetate and RRI. (**j**) Correlation analysis between content fold chang of Vitamin a and RRI. (**k**) Correlation analysis between content fold chang of All e-teprenone and RRI. (**l**) Correlation analysis between content fold chang of 5-amino-6-(5-phospho-beta-d-ribosylamino)uracil and RRI. (**m**) Correlation analysis between content fold chang of 12-aminolauric acid and RRI. (**n**) Correlation analysis between content fold chang of Steviol-19-o-glucoside and RRI. (**o**) Correlation analysis between content fold chang of Kukoamine b and RRI. (**p**) Correlation analysis between content fold chang of Farrerol and RRI.

Based on the above findings, we analyzed the correlation between the relative disease resistance index and the relative amount of this metabolite in the tree after *N. laricinum* infestation. Only 16 different metabolites showed a significant increase in the relative content of disease resistance (Figure 3). These included the oryzalin metabolite, spiromesifen, 9(R)-hexahydrocannabinol acetate, vitamin A, 5-amino-6-(5-phospho-beta-d-ribosylamino) uracil, kukoamine b, and farrerol, and there was a significant positive correlation between the levels of these seven metabolites in the tree and the relative disease resistance index (r > 0.5, *p* < 0.05). The correlation coefficients, in descending order, were as follows: farrerol (0.774), 9(R)-hexahydrocannabinol acetate (0.759), 5-amino-6-(5-phospho-beta-d-ribosylamino) uracil (0.661), vitamin a (0.647), kukoamine b (0.615), the oryzalin metabolite (0.599), and spiromesifen (0.571). The results showed that the positive correlation index between farrerol and the relative disease resistance index was higher and highly significant compared to the other seven metabolites.

### 2.3. WGCNA Screened 32 Genes Significantly Related to Farrerol

**Figure 4:**
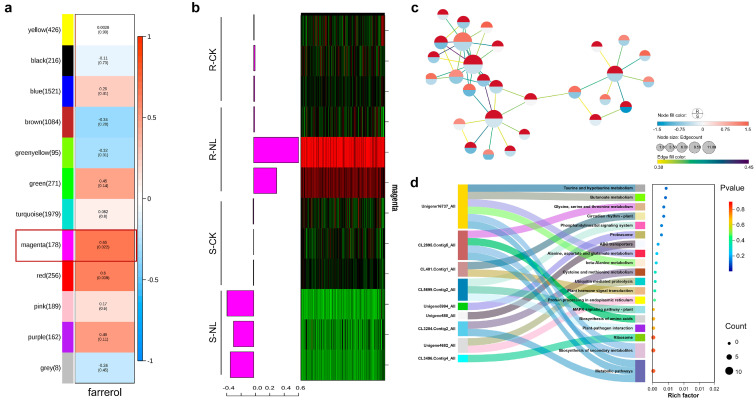
WGCNA analysis. (**a**) Module-trait correlation analysis. (**b**) Gene expression patterns within the magenta module and characterized gene expression patterns across samples. (**c**) Gene network map within magenta module. (**d**) KEGG enrichment analysis of genes in network map within magenta module.

A total of 12 distinct gene co-expression modules were identified through weighted gene co-expression network analysis. The interactions between these modules were subsequently analyzed using heat mapping, with each group of highly correlated genes corresponding to a branch of the tree. There was a high degree of consistency in gene expression patterns within the same module. The bright blocks of color along the diagonal indicated a high degree of topological overlap between the genes within the same module. The module that was significantly correlated with farrerol content was the magenta module (correlation coefficient 0.65, *p* = 0.022), which contained 178 genes (Figure 4a). Genes within this module were significantly up-regulated after *N. laricinum* infection in resistant larch (Figure 4b). A total of 32 genes (weight value > 0.40) were highly correlated with farrerol content, and an additional 32 genes were also correlated (weight value > 0.40) (Figure 4c).

KEGG enrichment analysis was performed on 32 genes, and a total of 19 pathways were enriched, belonging to 12 subcategories under 9 categories (Figure 4d), which were Amino acid metabolism; Carbohydrate metabolism; Environmental adaptation; Folding, sorting and degradation; Global and overview maps; Membrane transport; Metabolism of other amino acids; Signal transduction; and Translation.

### 2.4. Inhibitory Effect of Farrerol on N. laricinum

The results showed that the inhibitory effect of farrerol on *N. laricinum* increased with increasing concentrations of farrerol, indicating that farrerol as a phytochemical has the potential to inhibit pathogenic fungi, mainly in disease-resistant larch.

**Figure 5:**
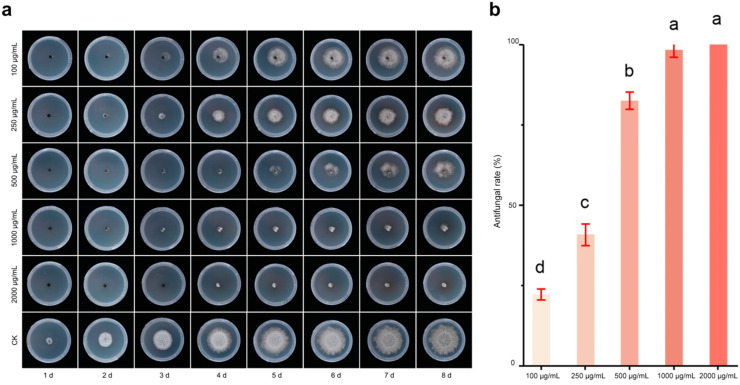
Antifungal effect of different concentrations of farrerol on *N. laricinum*. (**a**) Fungal inhibition effect of farrerol. (**b**) Fungal inhibition rate at 8 d after treatment with different concentrations of farrerol (one-way ANOVA).

When incubated for 8 days, 0.10 g/L, 0.25 g/L, 0.5 g/L, 1 g/L, and 2 g/L of farrerol showed antifungal activity, and the inhibition rate of 2 g/L was close to 100%. The mycelium of the control group mostly grew to the edge of the plate on day 6, while that of the treatment group continued to grow normally until 6 days later, when it gradually stopped growing (Figure 5a). A concentration of 0.10 g/L already had an inhibitory effect, and the inhibition rate was 22.2% after 8 d; at 0.25 g/L, the inhibition rate was 40.8%; at 0.5 g/L, the inhibition rate was 82.6%, which was more than half of the inhibition; at 1 g/L, the inhibition rate was 98.3%; and at 2 g/L, the inhibition rate was 100% (values after 8 d for each concentration). There was no significant difference in the fungal inhibition effect between the concentrations of 1 g/L and 2 g/L (Figure 5b).

The regression equation for the toxicity of farrerol on *N*. *laricinum* was y = 0.3693x + 0.4037 with an R^2^ value of 0.647, and the IC_50_ value was 0.257 g/L. The results demonstrated that the inhibitory effect of farrerol on *N*. *laricinum* showed a gradual increase with increasing concentration.

## 3. Discussion

Larch species, especially *Larix olgensis*, play an important role in forest ecosystem services, resource management, and timber production in China [19]. This study highlights the potential of the secondary metabolite farrerol as a natural fungicide to suppress *N. laricinum*, an invasive larch disease in China. The results demonstrated that farrerol has remarkable antifungal properties and effectively inhibits the mycelial growth of pathogenic fungus, making it a promising compound for plant defense against larch shoot blight. The positive correlation between farrerol levels and disease resistance in larch suggests its potential as a biopesticide against pathogenic fungi. Our study identified significant differences in disease resistance between different larch cultivars, with resistant cultivars exhibiting a relatively high disease resistance index of 81%, while susceptible cultivars exhibited severe symptoms after inoculation. Furthermore, a functional enrichment analysis of genes revealed pathways associated with farrerol content and highlighted signaling transduction and biochemical processes associated with candidate gene modules; this provides insights into the molecular mechanisms underlying disease resistance in larch.

Thus, the results of this study provide fundamental information on the potential of farrerol as a natural antifungal agent and its role in improving disease resistance in larch plantations. Our study highlights the importance of breeding disease-resistant larch varieties and integrating farrerol into disease management strategies. The results of this study suggest that the use of farrerol may help to develop more sustainable forestry practices, reducing reliance on chemical fungicides and promoting healthier forest ecosystems. Moreover, the utilization of farrerol as a biocontrol agent aligns with the increasing emphasis on sustainable agricultural practices and the reduction in chemical inputs in forestry [20,21,22,23]. By identifying resistant cultivars and exploring the role of farrerol, this research addresses a significant gap in understanding larch shoot blight, which poses a considerable threat to larch plantations in China. The results contribute valuable insights that can guide future breeding and management strategies, focusing on natural compounds essential for preventing, controlling, and eradicating forest diseases [24].

It is worth noting that *Neofusicoccum laricinum* has not been widely studied in relation to larch trees [9], although other studies have investigated the impact of various pathogens on different tree species [25]. We extend this knowledge by focusing on the molecular mechanisms of disease resistance and the role of farrerol in inhibiting the pathogen fungi. This finding offers novel insights into the natural defense strategies employed by plants. The integration of metabolomic and gene expression analysis in our research enhances the understanding of disease resistance mechanisms, providing insights that complement and extend those gained from previous studies that focused on either metabolomics or genomics in isolation [26,27]. Consistent with numerous studies in plant pathology, our findings underscore the significance of secondary metabolites in plant defense mechanisms [28,29]. Previous research has identified several metabolites, such as flavonoids and terpenoids, with antifungal properties against a range of pathogens [30,31]. For example, studies on various plant species have shown that flavonoids can inhibit the growth of fungi such as Fusarium and Aspergillus, which is consistent with the findings on the antifungal activity of farrerol presented in this study.

The results also show that resistant and susceptible plant species show different responses, characterized by varying degrees of severity, following inoculation with a pathogenic strain *in vitro* [32]. Consequently, the use of farrerol as a natural fungicide advocates the use of naturally derived compounds, which is in line with the growing trend towards sustainable forestry practices [23,28], in contrast to previous studies that mainly emphasized the efficacy of conventional fungicide treatments [33,34]. The identification of larch cultivars with significant disease resistance provides practical insights for breeding programs by identifying cultivars with robust resistance to *N. laricinum*.

Furthermore, our research underscores the significance of employing advanced technologies and established standards in breeding resistant larch varieties to combat shoot blight diseases. This approach is vital for ensuring the establishment of healthy plantations that yield quality timber while also providing ecological benefits. Large-scale cultivation and breeding of resistant larch are essential for enhancing the prevention and management of *N. laricinum* and elucidating the molecular mechanisms underlying disease resistance in different resistant monocultures.

## 4. Materials and Methods

### 4.1. Materials

To elucidate the mechanism through which farrerol inhibits larch shoot blight, this study utilized two larch genotypes, NL2 and NL4. These genotypes were selected based on a previous study that evaluated the resistance of ten larch genotypes with varying degrees of susceptibility and resistance. In that study, NL2 exhibited the highest resistance, while NL4 was identified as the most susceptible genotype. NL2 and NL4 were provided by the State Key Laboratory of Forest Tree Genetic Breeding, Northeast Forestry University.

The strain of *N. laricinum*, HLJ001, was obtained from the Key Laboratory of Monitoring and Control of Exotic Forest Pests and Diseases in Heilongjiang Province, Northeast Forestry University, and was molecularly and morphologically identified as *N. laricinum*. This strain was isolated from infected *L. olgensis* in Shangzhi City, Heilongjiang Province.

### 4.2. Methods

#### 4.2.1. Verification of Disease Resistance 

The identification of disease resistance in larch was achieved by artificial inoculation, and individual larch plants suitable for studying the molecular mechanism of disease resistance were screened. The 1-year-old non-lignified new shoots from 2-year-old branches of 10~20-year-old larch trees were inoculated with the HLJ001 strain, with six replicates for each larch. The larch branches were cut from healthy larch trees of the current year. Each branch was approximately 20 cm long and was selected to ensure uniformity of size and health. The sample was rinsed, placed in a 50 mL centrifuge tube, filled with 30 mL of water, and incubated in a greenhouse (16 h light, 8 h dark) at 25 °C for 1 d. Before inoculation, the samples were thoroughly rinsed with 75% alcohol, followed by sterile water, and the surface of the branches was dried with sterile filter paper. The bark was then carefully removed from the branch sections at the inoculation sites to expose the cambium layer. A sterilized blade was used to puncture the middle position of the branch, and the experimental group was inoculated with 2 × 5 mm mycelium blocks of the strain at the wound and the bag sealed, while the control group was treated with ddH_2_O, following the established indoor inoculation protocol for larch blight, with each branch receiving one sample of inoculum at each site. The inoculated larch was cultured in the tissue culture room (16 h light, 8 h dark, 25 °C, relative humidity > 80%).

To compare the responses between NL2 and NL4, several factors were assessed, including dehydration, wilting, needle shedding, color changes in the branches and stems, and the size of the lesions at the inoculation sites. At the same time, according to the classification of diseased plants, the classification was based on the following standardized criteria: 0: healthy; I: branches and stems turn green, a small number of needles fall off; II: branches and stems are yellow-brown, half of the needles fall off, and the tip droops slightly; III: branches and stems are brown, most of the needles fall off, and the tip droops; IV: branches and stems are dark brown, the tip droops, and all the needles fall off except for a cluster of purple-gray dead needles at the tip. The stability of resistance was confirmed by the consistent expression of symptoms across 6 replicates for both NL2 and NL4.

(1) $$\begin{array}{c} DI=\left[\frac{0n_{0}+1n_{1}+2n_{2}+3n_{3}+4n_{4}}{4n}\right]\times 100 \end{array}$$

(2) $$\begin{array}{c} RRI=1-\left[\frac{DIx}{DIy}\right] \end{array}$$

where *n*_0_~*n*_4_ are the number of plants under the corresponding disease level, and *n* is the total number of plants investigated. *DIx* is the disease index of a single plant measured, and *DIy* is the disease index of the most severely affected plant.

#### 4.2.2. Larch Metabolomics Assay and Data Analysis

Secondary metabolites associated with fungal inhibition and disease resistance were screened by comparing metabolome variations in resistant and sensitive larch plants. Disease-resistant and sensitive annual branches were inoculated as described in Section 4.2.1, with ddH_2_O mock inoculation treatment used as a control group. One gram of the larch samples to be tested was collected and stored in liquid nitrogen at −80 °C and entrusted to the Beijing Genomics Institute (BGI) for non-targeted metabolome detection using LC-MS/MS technology. The LC-MS/MS data were processed using Compound Discoverer 3.3 (Thermo Fisher Scientific, USA), which mainly included a series of analyses such as peak extraction, peak alignment, and metabolite identification [35]. The data exported from Compound Discoverer were subjected to data pre-processing and quality control analyses by Meta-X.

Multivariate statistical analysis of metabolites was conducted between samples in groups and metabolites within samples in groups using the analytical statistical method of orthogonal projections to latent structures-discriminant analysis, OPLS-DA with supervised discriminant patterns. SIM-CA-P 14.1 software was used for OPLS-DA analysis and model testing [36]. The validity of the OPLS-DA model was evaluated using the parameters of fitness (R^2^Y, threshold > 0.5) and predictability (Q^2^, threshold > 0.5), OPLS-DA score plots and S-Plot, and variable importance in the projection, VIP. Metabolites with significantly increased relative content were selected for subsequent correlation analyses based on the following criteria: VIP ≥ 1; *t*-test significance threshold, *p* < 0.05; and fold change (FC), FC ≥ 1.2.

SPSS 26.0 was used to process the data, and the correlation between the relative content of the significantly increased metabolites and the relative disease resistance index of larch was analyzed. The Pearson correlation coefficient (r) was calculated, and the differential metabolites with the highest correlation with the relative disease resistance index were identified.

#### 4.2.3. Screening of Genes Associated with Farrerol Based on Weighted Gene Co-Expression Network Analysis

The WGCNA package in R software 4.1.2 was employed to construct a gene co-expression network, and the genes with high similarity with respect to changes in farrerol content were explored in depth. Gene co-expression networks were analyzed to construct co-expression modules for the gene expression data of 12 samples of resistant and sensitive *L. olgensis* in the infected group and the CK group. A gene dissimilarity matrix was constructed to measure the difference between two genes by comparing the expression between the two genes. Meanwhile, based on the dissimilarity between genes, a hierarchical clustering tree was constructed using hierarchical clustering, and co-expression modules were identified using the Dynamic Tree Cut package. The initial principal component of each module, i.e., the module eigengene (ME), was derived through principal component analysis. The ME reflects the overall expression level of all genes in the module; cluster analysis was performed on the ME, and Merge Cut Height = 0.25 was set to group modules with high similarity into one cluster. The gene modules related to farrerol content were then screened for farrerol content.

#### 4.2.4. Gene Functional Enrichment Analysis within Candidate Modules

The genes within the candidate modules that exhibited a high degree of correlation with farrerol were subjected to enrichment analysis. The most significant biochemical and signal transduction pathways involved in the genes within the candidate modules were identified through the use of KEGG pathway enrichment analysis. Following correction with multiple testing, a threshold of FDR (False Discovery Rate) ≤ 0.05 was employed, whereby pathways meeting this criterion were defined as significantly enriched in the candidate module genes.

#### 4.2.5. Antifungal Test

A specific quantity of farrerol was dissolved in dimethyl sulfoxide (DMSO) and subsequently added to the PDA medium, resulting in farrerol concentrations of 0.10 g/L, 0.25 g/L, 0.5 g/L, 1 g/L, and 2 g/L, respectively. A control group was established using the same volume of DMSO without the inclusion of farrerol. A 5 mm mycelium block of the *N. laricinum* larch fungus, selected with a perforator, was inoculated into the PDA medium at different concentrations, with five replicates for each treatment condition. The cultures were incubated at 25 °C in the dark, and the mycelial growth was assessed and photographed at 24 h intervals. Colony diameter was determined using the criss-cross method, and colony area and inhibition rate were calculated.

(3) $$I=\frac{{\pi \left(\frac{D_{1}-5}{2}\right)}^{2}-{\pi \left(\frac{D_{2}-5}{2}\right)}^{2}}{{\pi \left(\frac{D_{1}-5}{2}\right)}^{2}-{5\pi }^{2}}\times 100\%$$

where *I* represents the inhibition rate, *D*_1_ is the colony diameter of the control group, and *D*_2_ is the colony diameter of the treatment group.

## 5. Conclusions

This study provides fundamental information on the potential of farrerol as a natural antifungal agent and its role in improving disease resistance in larch plantations. The results highlight a promising approach to improving the health and productivity of larch trees in the context of increasing disease threats through the use of chemicals that are sustainable, environmentally non-toxic, residue-free, and beneficial to forest wildlife, including soil-dwelling organisms, as well as human health. The identification of resistant cultivars and the study of the genetic mechanisms underlying disease resistance provide practical insights for breeding programs, while the use of farrerol as a biocontrol agent provides a viable alternative to conventional fungicides, which often have a negative impact on the environment. This research provides valuable insights into plant science and disease management, promotes sustainable practices, and enhances our understanding of plant–pathogen dynamics. In summary, our studies serve as a cornerstone for future research endeavors aimed at elucidating the complexities of secondary metabolites on disease management and the potential for integrating these approaches into sustainable forestry practices.

## Figures and Tables

**Table 1:** The 16 screened compounds.

Index	Name
MATE1009	4-tert-butylcyclohexyl acetate
MATE1224	Ginkgolic acid c15:1
MATE1432	Disperse orange 3
MATE2126	Oryzalin metabolite
MATE2455	Idebenone
MATE261	D-(+)-camphor
MATE2651	Spiromesifen
MATE2777	(2e,6e)-farnesyl monophosphate
MATE3234	9(R)-Hexahydrocannabinol Acetate
MATE3380	Vitamin a
MATE3474	All e-teprenone
MATE3944	5-amino-6-(5-phospho-beta-d-ribosylamino)uracil
MATE4643	12-aminolauric acid
MATE853	Steviol-19-o-glucoside
MATE995	Kukoamine b
MATE23	Farrerol

## Data Availability

The RNA sequencing data have been deposited in the NCBI Sequence Read Archive (http://www.ncbi.nlm.nih.gov/sra, accessed on 22 October 2024) database.
